# Palmetto Wine

**Published:** 1895-08

**Authors:** Ira W. Porter

**Affiliations:** Late Health Officer of Sanford, Fla., Mobile, Ala.; No. 13 N. Conception Street


### PALMETTO WINE.

By IRA W. PORTER, M. D.,
Late Health Officer of Sanford, Fla., Mobile, Ala.

I wish to call the attention of the profession to a new preparation of a well-known drug, one that I have used with remarkable success. It is strictly, speaking, a southern preparation of a southern drug.

Palmetto wine is a new and elegant preparation of raw palmetto, and combines all the medicinal virtues of the drug in a palatable form.

It is best known as Palmetto Wine (Wellington) and is made and used in large quantities in Sanford, Florida, and the surrounding country. The menstruum is the elegant and pleasing orange wine, made from the famous Florida orange.

I have used it on children and on the aged, as a tonic, with good effect. Its fattening properties are astonishing.

I used it on a middle-aged man, who was suffering from nervous impotence, with very gratifying results.

In women I have used it to promote the growth of the mammæ, and internally, in gonorrhoea, I have used it with better results than with sanmetto.

It is also of use in bronchial troubles. The raw palmetto seems to have a peculiar action on all mucous surfaces, and has been used with gratifying effect in bronchitis laryngitis, follicular pharyngitis, tonsilitis, etc.

This reads somewhat like an advertisement, but such is not the intention of the writer, as he has no personal interest in the manufacture or sale of the wine.

I have used it for over a year with so much satisfaction, that I felt I ought to make such a preparation known to the profession.

No. 13 N. Conception Street.

